# The National Library of Medicine’s Disaster Information Management Research Center

**DOI:** 10.3389/fpubh.2013.00070

**Published:** 2013-12-16

**Authors:** Steven J. Phillips

**Affiliations:** ^1^National Library of Medicine, National Institutes of Health, Bethesda, MD, USA

**Keywords:** library, informationist, literature, tools, apps

## Abstract

The Disaster Information Management Research Center (DIMRC) develops and provides access to health information resources and technology for disaster preparedness, response, and recovery. DIMRC focuses on maintaining access to health information at all phases of disasters, developing innovative products and services for emergency personnel, conducting research to support disaster health information management, and collaborating with other agencies and communities. Several tools are available to help emergency responders in hazardous materials or chemical, biological, radiological, or nuclear incidents. Access to the literature is made available through PubMed and the Resource Guide for Disaster Medicine and Public Health, with links to online documents and resources from numerous organizations and government agencies. In addition, DIMRC supports the Disaster Information Specialist Program, a collaborative effort to explore and promote the role of librarians and information specialists in the provision of disaster-related information resources to the workforce and communities.

The National Library of Medicine (NLM), in Bethesda, MD, USA, is part of the National Institutes of Health. NLM has been a center of information innovation since it’s founding in 1836. The NLM is the world’s largest biomedical library that maintains and makes available a vast print collection and produces electronic information resources on a wide range of topics that are searched billions of times each year by millions of people around the globe. NLM supports and conducts research, development, and training in biomedical informatics and health information technology and coordinates a 6,000-member National Network of Libraries of Medicine that promotes and provides access to health information in communities across the United States.

National Library of Medicine specific coverage of disaster information dates from the late 70s, when disaster planning and relief were recognized as medical subject headings (1) terms. (MeSH). NLM increased its involvement in disaster management by providing bibliographic services in Central America following Hurricane Mitch (1998) and by increasing and referencing disaster topics in MEDLINE. The information gap in the aftermath of Hurricane Katrina provided the trigger for a strengthening of NLM’s mandate in this field and allowing the institution to apply domestically the experience gained in the establishment of Central American Network for Disaster and Health Information (2), or CANDHI.

The term “disaster” covers events of many types: natural disasters, technological disasters, and acts of violence (Table 1). It is universally recognized that information and communication are key components in all aspects of disaster management. For example, the importance of information is emphasized in government publications such as the Emergency Support Function (ESF) #8 (3) and the Pandemic and All Hazards Preparedness Act emphasize (4) (PAHPA).

**Table 1:** **Disaster types**.

Bioterrorism
Chemical emergencies
Fires and wildfires
Geological: earthquakes
Pandemic disease outbreaks
Radiation emergencies
Weather and storms: floods

No single US Government agency is missioned specifically to collect, organize, provide, and communicate disaster health information. NLM responded to Congress (5, 6) for that need in 2008 by creating a Disaster Information Management Research Center (DIMRC). As part of NLM’s Specialized Information Services (SIS) Division, DIMRC coordinates all of NLM disaster-related activities and is tasked with the collection, organization, and dissemination of health information resources and informatics research related to disasters of deliberate, natural, or accidental origin.

Examples of DIMRC activities include:

Disaster Literature Initiative^1^
  ○ The Resource Guide for Disaster Medicine and Public Health is a gateway to freely available online resources related to disaster medicine and public health with links to over 3,000 publications, expert guidelines, reports, websites, and fact sheets, research reports, articles, and other tools aimed at the public health community.
  ○ Enviro-Health Links series with a dozen guides on disaster topics for health professionals
  ○ MedlinePlus has nearly 40 subject pages on all-hazards topics for the public, in English and Spanish
  ○ PubMed has more than 40,000 medical journal articles on disaster topics from 5,000 journals, including 20+ journals exclusively on disaster and emergency medicine
  ○ Disaster apps and mobile optimized web pages^2^
  ○ Emergency Access Initiative (EAI) provides temporary free access to full text articles from major biomedicine titles to healthcare professionals, librarians, and the public affected by disasters^3^.
Disaster Information Specialist Project^4^ is an effort to develop a new subspecialty in library science: the Disaster information Specialist Librarian (DIS). The DIS is the information specialist who in many locations, including Washington, DC, is part of the emergency preparedness and response team.
  ○ The Medical Library Association with the NLM has developed basic and advanced courses to train librarians and other interested professionals as disaster information specialists
  ○ Disaster-Outreach-Lib Listserv email discussion forum covering 50 US states and 10 countries includes librarians, information specialists, and other professionals interested in disaster health information outreach to their communities.
Responder and receiver decision support tools: utilizing NLM’ s Hazardous Substances Databank (7), DIMRC has developed several emergency responder and receiver decision support tools to prepare for and respond to chemical, biological, radiological, and nuclear events (CBRN). These Tools are accessible and downloadable from our web sites, or as an App for a mobile device.
  ○ Wireless Information for Emergency Responders, or WISER^5^, is a system designed to assist first responders in hazardous material incidents and biological hazards (Table 2). WISER provides a wide range of information on hazardous substances, including substance identification support, information to aid in responding to hazardous materials incidents, including emergency medical treatment, protective distance maps, and personal protective equipment needed by the responders. WISER contains
  ○ REMM^6^, the Radiation Emergency Medical Management, offers guidance and algorithms to healthcare professionals, and responders on diagnosis, management, countermeasures, and treatment during radiation events such as a nuclear explosion, dirty bomb, or nuclear accident.
  ○ Chemical Hazards Emergency Management or CHEMM^7^ is designed to assist emergency responders and health care providers in the management of large-scale chemical incidents.
Patient Information Management Research Projects
  ○ Data Capture by a Digital Pen (8) that records disaster patient triage data and uplinks the data to a computer database for use at the receiving hospital.
  ○ Tracking Patients with radio frequency identification (RFID) tags in a real-time location system (RTLS) (9, 10)
  ○ Lost Person Finder/ReUnite project^8^ uses photographic information technology to enable family, friends, and neighbors to locate missing patients during a disaster
  ○ Military Affiliate Radio System (MARS) is being evaluated as a back-up communications system, including a prototype method of sending digital data (11).
  ○ Virtual World Interactive Disaster Training utilizing traditional multimedia instruction with “virtual world” technologies for disaster preparedness training^9^.
Bethesda Hospitals’ Emergency Preparedness Partnership: Disaster Information Management Research Center serves as a focal point for NLM’s participation in the. BHEPP, established in 2004 with Congressional earmarks, is a partnership between the Walter Reed National Military Medical Center, National Institutes of Health Clinical Center, Suburban Hospital Johns Hopkins Healthcare System, and the NLM (12, 13) (Table 3).

**Table 2:** **Bioterrorism agents/diseases**.

Anthrax
*Bacillus anthracis*
Botulism
*Clostridium botulinum* toxin
*Francisella tularensis*
Plague
Small pox
Tularemia
*Variola major*
VHF
Viral hemorrhagic fevers
*Yersinia pestis*

**Table 3:** **BHEPP disaster research and development projects**.

Patient information management	Communications	Information access	Responder training
Digital pen	Laser back-up	Prescription drug access in disaster	Virtual world disaster training
Patient data exchange	Dark fiber back-up	Disaster information specialist	
Patient RFID tracking	MARS radio back-up	Lost person finder	
	Wireless voice bridge		

BHEPP’s main goals include:

○ Use the combined resources of the partnership to respond rapidly and successfully to a National Capital emergency hospital surge needs.
○ Develop an exportable BHEPP Hospital Surge Model to centers across the Nation.
○ Research, Develop and test collaborative structures, processes and tools to support the partnership, responders and receivers.

## Summary

Access to vital health information is a common need of communities, individuals, and government agencies for disaster preparedness, response, mitigation, and recovery. To help achieve that goal, in 2008, NLM established a one of a kind DIMRC to specifically to work with DHHS, other lead agencies and with the disaster medicine and emergency management communities^10^ to better meet their critical information needs related to local, regional, national and international emergency preparedness, response, mitigation, and recovery. DIMRC at NLM has demonstrated how libraries and librarians can be part of the solution to disaster remediation.

## Conflict of Interest Statement

The author declares that the research was conducted in the absence of any commercial or financial relationships that could be construed as a potential conflict of interest.

## Footnotes

^1^http://sis.nlm.nih.gov/dimrc/medscilit.html

^2^http://sis.nlm.nih.gov/dimrc/disasterapps.html

^3^http://eai.nlm.nih.gov/docs/captcha/test.pl?url=

^4^http://sis.nlm.nih.gov/dimrc/disasterinfospecialist.html

^5^http://wiser.nlm.nih.gov/

^6^http://www.remm.nlm.gov/

^7^http://chemm.nlm.nih.gov/

^8^https://pl.nlm.nih.gov/

^9^http://infocus.nlm.nih.gov/2013/06/virtual-training-for-real-worl.html

^10^http://sis.nlm.nih.gov/dimrc/partnersdimrc_ppp.html
